# Is Bulpa criteria suitable for the diagnosis of probable invasive pulmonary Aspergillosis in critically ill patients with chronic obstructive pulmonary disease? A comparative study with EORTC/ MSG and ICU criteria

**DOI:** 10.1186/s12879-017-2307-y

**Published:** 2017-03-14

**Authors:** Linna Huang, Hangyong He, Jingjing Jin, Qingyuan Zhan

**Affiliations:** 1 Department of Pulmonary and Critical Care Medicine, Centre for Respiratory Diseases, China-Japan Friendship Hospital, Beijing, People’s Republic of China; 2 Beijing Institute of Respiratory Medicine, Department of Respiratory and Critical Care Medicine, Beijing Chao-Yang Hospital, Capital Medical University, Beijing, People’s Republic of China; 3 Department of Critical Care Medicine, Children’s Hospital affiliated with the Capital Institute of Pediatrics, Beijing, People’s Republic of China; 4 No.2 Yinghua East Road, Chaoyang District, Beijing, 100029 China

**Keywords:** Invasive pulmonary aspergillosis, Chronic obstructive pulmonary disease, Bulpa criteria, EORTC/ MSG criteria, ICU criteria

## Abstract

**Background:**

Three diagnostic criteria have been proposed used for invasive pulmonary aspergillosis (IPA) diagnosis, namely EORTC/ MSG criteria, Bulpa criteria and intensive care unit (ICU) criteria. The Bulpa criteria were proposed to diagnose IPA in chronic obstructive pulmonary disease (COPD) patients specially. Our aim is to verify that whether the Bulpa criteria are the most suitable for diagnosing probable IPA in critically ill COPD patients compared with the other two criteria.

**Methods:**

We included critically ill COPD patients admitted to the ICU from April 2006 to August 2013. Patients were classified into four populations: population one (n_1_ = 59) comprised all included patients; population two (n_2_ = 24) comprised patients with positive mycological findings (both positive cultures and positive serologic tests); population three (n_3_ = 18) comprised patients with positive lower respiratory tracts (LRTs) isolation; and population four (n_4_ = 5) comprised proven IPA patients with histopathology. Patients in four groups were diagnosed as probable IPA using three criteria respectively, and the “diagnostic rate” of each criteria were compared with each other. Then, the reasons for differences in “diagnostic rate” were analyzed in population two. Finally, the modified Bulpa criteria were proposed.

**Results:**

Bulpa criteria yielded the highest “diagnostic rate” of probable IPA followed by the ICU criteria, while the EORTC/ MSG criteria provided the lowest rates in four populations (the “diagnostic rate” of probable IPA was 33.9%, 16.9% and 6.8% in population one, *p* = 0.001; 83.3%, 41.7% and 16.7% in population two, *p* < 0.001; 100%, 55.6% and 22.2% in population three, *p* < 0.001; 100%, 60% and 20% in population four, *p* = 0.036). The reasons for the highest “diagnostic rate” by Bulpa criteria were its less strict requirements regarding the doses/courses of steroid use and typical computed tomography (CT) findings. Finally, the modified Bulpa criteria for probable IPA were proposed for critically ill COPD patients admitted to ICU, mainly involving revised interpretations of microbiological findings.

**Conclusions:**

Among the existing three criteria, the Bulpa criteria are the most suitable for diagnosing probable IPA in critically ill COPD patients admitted to ICU. A modified criteria maybe proposed for better diagnosis,and its clinical validity need to be verified in future studies.

**Electronic supplementary material:**

The online version of this article (doi:10.1186/s12879-017-2307-y) contains supplementary material, which is available to authorized users.

## Background

Chronic obstructive pulmonary disease (COPD) has been recognized as a potential risk factor of invasive pulmonary aspergillosis (IPA) [1–3]. The average mortality of IPA in COPD patients has been found to be as high as 50-100% [1, 4, 5], and is especially in patients with delayed diagnosis.

Tremendous challenges in early diagnosis of IPA existed among specialists in pulmonary and critical care medicine. Therefore, appropriate diagnostic criteria are crucial for IPA at the early stage.

Three diagnostic criteria have been proposed used for IPA diagnosis. The EORTC/ MSG criterion was proposed by a consensus group of the European Organization for the Research and Treatment of Cancer/Invasive Fungal Infections Cooperative Group (EORTC) and the National Institute of Allergy and Infectious Diseases Mycoses Study Group (MSG) in the year 2002 for use with haematological patients, and this criteria were revised in 2008 [6, 7]. Bulpa proposed another set of criteria in 2007 specifically for use with COPD patients in respiratory ward [8]. According to both sets of criteria, IPA is categorized as proven, probable or possible. A third set of criteria was put forwarded by Vandewoude use with intensive care unit (ICU) patients, with an entry criterion of positive lower respiratory tract (LRT) specimens for *Aspergillus*; this set categorized IPA as proven or putative, where putative IPA could be regarded as probable IPA in the first two criteria sets mentioned above [9, 10]. Since the critically ill COPD patients admitted to ICU had their particular characteristics which were huge different with severe immunocompromised patients, we made a clinical assumption that Bulpa criteria are the most suitable for diagnosing probable IPA in critically ill COPD patients admitted to ICU compared with the other two criteria.

## Methods

### Study population and inclusion criteria

All of the patients were admitted to our respiratory ICU (RICU) due to respiratory failure from April 2006 to August 2013.

#### Inclusion criteria

We included patients based on the following criteria:

1. Older than 50 years old.
2. Severe COPD with a pulmonary functional level of stage III or IV according to the Global Initiative for Chronic Obstructive Lung Disease (GOLD). We made the corresponding changes based on the updated GOLD guidelines [11–13].
3. At least one computed tomography (CT) recorded during their hospitalization. CT(s) were provided within 1 week prior to admission to the RICU.

#### Exclusion criteria

We excluded patients based on the following criteria:

1. Patients with solid organ transplantation (SOT) and haematopoietic stem cell transplantation (HSCT), which included allergenic and autologous HSCT.
2. Patients with neutropenia, blood system diseases, malignant tumors and human immunodeficiency virus (HIV) infection.
3. Patients who accepted a high dosage of immunosuppressant because of connective tissue disease (CTD) in the previous 3 months.

### Diagnostic criteria

#### Definitions for proven IPA

Items required for proven IPA were same in the three criteria [7–10].

Microscopic analysis of sterile material: histopathological, cytopathological, or direct microscopic examination of a specimen obtained by needle aspiration or sterile biopsy in which hyphae are observed and are accompanied by evidence of associated tissue damage.

Culture on sterile material: recovery of *Aspergillus* by culturing of a specimen obtained by lung biopsy.

#### Definitions for Probable/Putative IPA

The definition for probable/ putative IPA can be concluded based on the following three types of information: host factors, clinical data and microbiological findings.

The following is a brief description of the three parts in each diagnostic criterion (Table 1). The main differences among three criteria were marked with italic and underline.

**Table 1:** Brief descriptions of each diagnostic criteria

	EORTC/ MSG Criteria	Bulpa Criteria	ICU Criteria
*Host factors*	**i)** A recent history of neutropenia (<0.5*10^9^ neutrophils/L for more than 10 days) that is temporally related to the onset of fungal disease. **ii)** Receipt of an allogeneic stem cell transplant. **iii)** Prolonged use of *corticosteroids at a mean minimum dose of 0.3 mg/kg/day of a prednisone equivalent for more than 3 weeks.* **iv)** Treatment with other recognized T cell immunosuppressants, such as cyclosporine, TNF-α blockers, specific monoclonal antibodies or nucleoside analogues during the previous 90 days. **v)** Inherited severe immunodeficiency.	**i)** Patients with a pulmonary functional level of stage III or IV according to the GOLD guidelines. **ii)** Patients *treated with steroids, with no strict requirement regarding the usage, dosage or duration*.	**i)** Neutropenia (absolute neutrophil count < 500/mm^3^) preceding or at the time of ICU admission. **ii)** Underlying haematological or oncological malignancy treated with cytotoxic agents. **iii)** *Glucocorticoid treatment (prednisone equivalent, >20 mg/d).* **iv)** Congential or acquired immunodeficiency.
*Clinical data*	Patients must have subjected to *at least one CT scan and must exhibit 1 of the following 3 signs*: **i)** Dense, well-circumscribed lesion(s) with or without a halo sign. **ii)** An air-crescent sign. **iii)** A cavity.	**i)** Patients with recent exacerbation of dyspnea despite the administration of appropriate antibiotics. **ii)** Patients with *progressive deterioration of chest imaging findings* (within three months)	**i)** One of the following compatible signs or symptoms: ① Fever refractory to at least three days of appropriate antibiotic therapy. ② Recrudescent fever after a period of defervescence of at least 48 h while still on antibiotics and without other apparent cause. ③ Pleuritic chest pain. ④ Pleuritic rub. ⑤ Dyspnea. ⑥ Haemoptysis. ⑦ Worsening respiratory insufficiency despite appropriate therapy and ventilator support. **ii)** *Patients with abnormal chest X rays (CXRs) or CTs.*
*Mycological findings*	**i)** Positive culture and/or microscopy result for *Aspergillus* from the lower respiratory tracts (LRTs). **ii)** Positive *serum or bronchoalveolar lavage fluid (BALF) galactomannan (GM) tests.*	**i)** Positive culture and/or microscopy findings for *Aspergillus* from the LRTs. **ii)** Positive *serum antibody test* for *A. fumigatus* (including precipitin). **iii)** *Two consecutive positive serum GM tests.*	Positive culture for *Aspergillus* from the LRTs.

Note: We concluded each criteria as three parts, namely the host factors, clinical data and microbiological findings and marked the main differences among three criteria italic and underline. *EORTC/MSG* European Organization for Research and Treatment of Cancer/Invasive Fungal Infections Cooperative Group and the National Institute of Allergy and Infectious Diseases Mycoses Study Group; *ICU* Intensive Care Unit; TNF-α: Tumor Necrosis Factor-α; *CT* Computed Tomography; *CXR* Chest X Ray; *LRT* Lower respiratory tract; *BALF* Bronchoalveolar Lavage Fluid; *GM* Galactomannan

Additional instructions in EORTC/ MSG Criteria [7]:

Because of the exclusion criteria adopted in our study, our patients may only satisfy condition iii).

Critically ill COPD patients who met host factor iii), one of the clinical data and one of the microbiological findings were diagnosed as probable IPA.

Additional instructions in Bulpa Criteria [8]:

In our hospital, the detection of serum antibody or precipitin for A. fumigatus was unavailable.

The cut-off values of the serum galactomannan (GM) and broncho-alveolar lavage fluid (BALF) GM tests are 0.5 ng/ml and 0.8 ng/ml [14, 15], respectively. Serum GM levels greater than 0.5 ng/ml and BALF GM levels greater than 0.8 ng/ml were defined as positive results.

Critically ill COPD patients who met all host factors, all clinical criteria and one of the microbiological findings were diagnosed as probable IPA.

Additional instructions in ICU Criteria [9, 10]

If the patients did not present any host factors, a semi-quantitative *Aspergillus*-positive culture of BALF (+ or ++), without bacterial growth together but with a positive cytological smear showing branching hyphae is considered as alternative. Unfortunately, the semi-quantitative *Aspergillus* culture of BALF was not routinely available at our institution during the study period.

The same as the EORTC criteria, because of the exclusion criteria adopted in our study, our patients may only present condition iii).

Critically ill COPD patients who met host factor iii), all clinical data and microbiological findings were diagnosed as putative IPA.

### Data collection and analysis

Patient characteristics, including age, sex, the usage, dosage and duration of steroids prior toadmission to the RICU, microbiological examinations (including cultures of the sputum, endotracheal aspiration (ETA) and BALF, and serum GM and BALF GM tests), chest radiological data (chest X rays (CXRs) and CT scans) and clinical outcomes were collected.

Two experienced respiratory physicians and a radiologist who were not aware of the clinical outcomes of the patient analysed the radiological data.

#### Methods for population divisions

All patients were classified into four populations. The scope of each group gradually reduced.

Population one comprised all included COPD patients; population two comprised patients with positive mycological findings (both positive cultures and positive serologic tests); population three comprised patients with the positive LRTs isolation, which including sputum, ETA and BALF; and population four comprised proven cases of IPA based on histopathology.

Usually, the validity of diagnostic criteria is assessed according to a gold standard, which depends on the use of biopsy or necropsy. However, it was difficult to obtain tissue specimen for critically ill COPD patients; therefore, we evaluated the “diagnostic rate” of three criteria in different populations, and verified the result based on a proven IPA population.

Patients from each population were diagnosed as probable/ putative IPA using the above criteria, and the “diagnostic rate” were compared among them. Patients who did not fall into the probable/ putative IPA group were considered as non-classifiable. The reasons for differences in the “diagnostic rate” among the three criteria were then analysed in population two (the population with positive mycological findings). Finally, the modified Bulpa criteria for probable IPA in critically ill COPD patients who have been admitted to ICU were proposed.

### Statistical analysis

Our data were categorical variables; therefore, comparative analyses were performed using a Pearson Chi-Square (*x* ^2^ test). For sample sizes of less than 40 or theoretical frequencies (T) of less than 1, Fisher’s Exact Test was used. P values < 0.05 were considered to indicate statistical significance.

## Results

From April 2006 to August 2013, 171 COPD patients were admitted to the RICU because of respiratory failure. The numbers of patients who suffered from malignancy, used a high doses of immunosuppressant in the previous 3 months or for whom integrated radiological imaging was not available were 3,6,2 and 101, respectively; these patients were excluded from the study. 59 patients who met the inclusion criteria were enrolled.

**Fig. 1:**
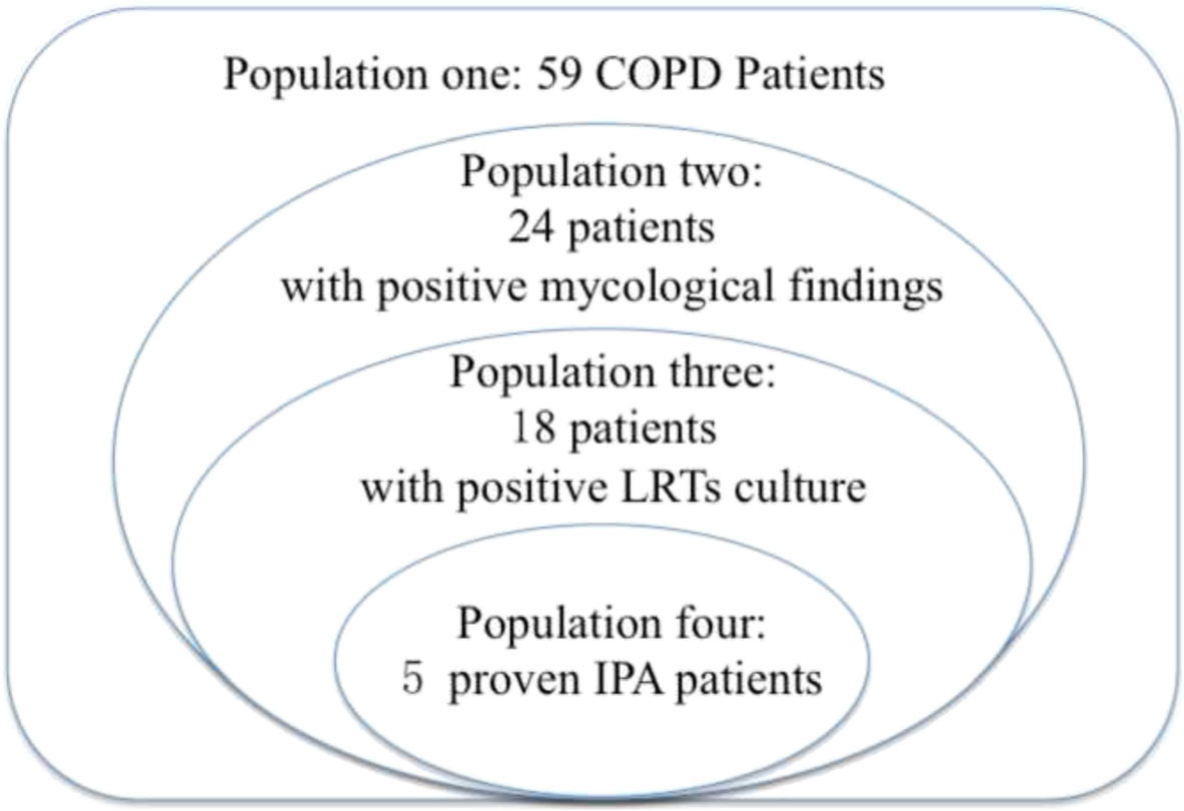
All 59 patients were classified into four populations. Population one comprised all 59 included patients; population two comprised 24 patients with positive mycological findings (both positive cultures and positive serologic tests); population three comprised 18 patients with the positive LRTs isolation; and population four comprised 5 proven cases of IPA based on histopathology

All 59 critically ill COPD patients were classified into four populations according to the previously described methods. Population one included all 59 critically ill COPD patients; population two included 24 patients with positive mycological findings (both positive cultures and positive serologic tests); population three included 18 patients with positive LRTs isolation; and population four included 5 proven IPA cases based on bronchial mucosal biopsy (Fig. 1).

### General information

The history of steroids use, typical CT manifestations and microbiological findings of the patients were provided in Additional file 1.

### Estimate of the “ Diagnostic rate” for the three criteria in each population

The patients in the four populations were diagnosed as probable/ putative IPA using the Bulpa criteria, ICU criteria and EORTC/ MSG, which were recorded in Table 2. The respect “diagnostic rates” were 33.9%, 16.9% and 6.8% in population one, p = 0.001; 83.3%, 41.7% and 16.7% in population two, p < 0.001; 100%, 55.6% and 22.2% in population three, p < 0.001; 100%, 60% and 20% in population four, p = 0.036. The Bulpa criteria resulted in the highest “diagnostic rate” of probable IPA, followed by the ICU criteria and the EORTC/ MSG criteria, in that order; this trend was confirmed by population four (the proven IPA patients).

**Table 2:** The “ Diagnostic rate” in different populations according to three criteria

Diagnostic rate n (%)	Bulpa Criteria	ICU Criteria	EORTC/MSG Criteria	*P* value
Population one (*n* = 59)	20 (33.9)	10 (16.9)	4 (6.8)	0.001*
Population two (*n* = 24)	20 (83.3)	10 (41.7)	4 (16.7)	<0.001*
Population three (*n* = 18)	18 (100)	10 (55.6)	4 (22.2)	<0.001*
Population four (*n* = 5)	5 (100)	3 (60)	1 (20)	0.036*

Note: Population one: all included COPD patients; population two: patients with positive mycological findings; population three: patients with positive lower respiratory tracts (LRTs) isolation; population four: proven IPA patients. The “diagnostic rate” of probable IPA were 33.9%, 16.9% and 6.8% in population one, *p* = 0.001; 83.3%, 41.7% and 16.7% in population two, *p* < 0.001; 100%, 55.6% and 22.2% in population three, *p* < 0.001; 100%, 60% and 20% in population four, *p* = 0.036. *EORTC/MSG* European Organization for the Research and Treatment of Cancer/Invasive Fungal Infections Cooperative Group and the National Institute of Allergy and Infectious Diseases Mycoses Study Group; *ICU* Intensive Care Unit; *: *P* < 0.05

### Analysis of the reasons for the non-classification of patients by each set of diagnostic criteria

The findings regarding “diagnostic rate” followed the trend: Bulpa criteria, ICU criteria and EORTC/ MSG criteria; therefore, we attempted to determine the reasons for these differences by comparing any two criteria in population two.

#### Comparison between EORTC/ MSG criteria and Bulpa criteria

**Fig. 2:**
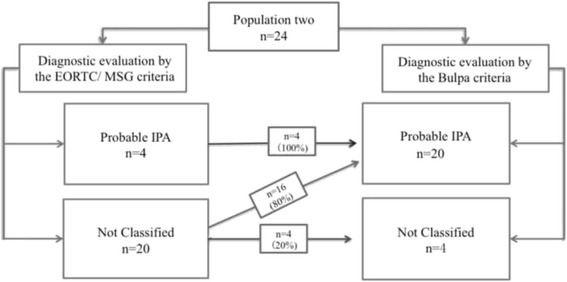
Comparison between the EORTC/ MSG and Bulpa criteria. We found that all probable IPA diagnosed by the EORTC/ MSG criteria were also diagnosed by the Bulpa criteria; 16 (80%) patients who were not classified by EORTC could be diagnosed as probable IPA by the Bulpa criteria, and the remaining 4 (20%) patients remained non-classified even based on the Bulpa criteria

We found that all probable IPA diagnosed by the EORTC criteria were also diagnosed by the Bulpa criteria; 16 (80%) patients who were not classified by EORTC/ MSG could be diagnosed as probable IPA by the Bulpa criteria, and the remaining 4 (20%) patients remained non-classified even based on the Bulpa criteria (Fig. 2).

#### Comparison between ICU criteria and Bulpa criteria

**Fig. 3:**
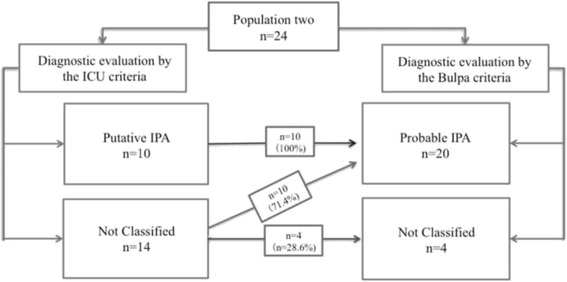
Comparison between the ICU and Bulpa criteria. We found that all cases that were diagnosed as probable IPA by the ICU criteria were also diagnosed by the Bulpa criteria; 10 (71.4%) cases that were not classified by ICU were diagnosed as probable IPA by the Bulpa criteria, and the remaining 4 (28.6%) patients remained non-classified even based on the Bulpa criteria

We found that all cases that were diagnosed as probable IPA by the ICU criteria were also diagnosed by the Bulpa criteria; 10 (71.4%) cases that were not classified by ICU were diagnosed as probable IPA by the Bulpa criteria, and the remaining 4 (28.6%) patients remained non-classified even based on the Bulpa criteria (Fig. 3).

#### Comparison between EORTC/ MSG criteria and ICU criteria

**Fig. 4:**
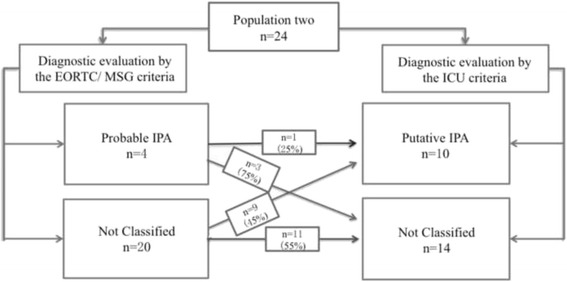
Comparison between the EORTC/ MSG and ICU criteria. We found that only 1 (25%) case that was diagnosed as probable IPA based on the EORTC/ MSG criteria was diagnosed by the ICU criteria, and the remaining 3 (75%) cases were not classified; 9 (45%) cases that were not classified by EORTC/ MSG were diagnosed as probable IPA based on the ICU criteria, and the remaining 11 (55%) patients remained non-classified even by the ICU criteria

We found that only 1 (25%) case that was diagnosed as probable IPA based on the EORTC/ MSG criteria was diagnosed by the ICU criteria, and the remaining 3 (75%) cases were not classified; 9 (45%) cases that were not classified by EORTC were diagnosed as probable IPA based on the ICU criteria, and the remaining 11 (55%) patients remained non-classified by the ICU criteria (Fig. 4).

#### The reason underlying the non-classification of patients by the EORTC/ MSG criteria

Among 24 patients with positive mycological findings, only 4 were diagnosed as probable IPA cases; the non-classification of 20 patients was attributed to the strict requirements regarding the dosage and duration of steroid use and the typical CT findings (Table 3).

**Table 3:** Reasons for the non-classification of patients by the three criteria in population two

Reason for diagnosis failure	EORTC/ MSG Criteria (*n* = 20)	Bulpa Criteria (*n* = 4)	ICU Criteria (*n* = 14)
Dose of steroid	0 (0%)	0 (0%)	9 (64.3%)
Course of steroid	6 (30%)	0 (0%)	0 (0%)
Dose and course of steroid	2 (10%)	0 (0%)	0 (0%)
Typical CT findings	1 (5%)	0 (0%)	0 (0%)
Steroid and CT	11 (55%)	0 (0%)	0 (0%)
BALF GM	0 (0%)	4 (100%)	0 (0%)
Positive LRTs culture	0 (0%)	0 (0%)	1 (7.1%)
Steroid and LRTs culture	0 (0%)	0 (0%)	4 (28.6%)

Note: the non-classification of patients by each criteria were listed above; the main reasons were the strict requirements regarding steroid use, typical CT findings and positive LRTs cultures

*EORTC/MSG* European Organization for Research and Treatment of Cancer/Invasive Fungal Infections Cooperative Group and the National Institute of Allergy and Infectious Diseases Mycoses Study Group; *ICU* Intensive Care Unit; *CT* Computed Tomography; *BALF* Bronchoalveolar Lavage Fluid; *GM* Galactomannan; *LRT* Lower respiratory tract

#### The reason underlying the non-classification of patients by the Bulpa criteria

Among 24 patients with positive mycological findings, 20 were diagnosed as probable IPA cases; the non-classification of the remaining 4 patients was attributed to the lack of consecutive BALF GM in the mycological findings of the Bulpa criteria (Table 3).

#### The reason underlying the non-classification of patients by the ICU criteria

Among 24 patients with positive mycological findings, 10 were diagnosed as putative IPA cases; the non-classification of the remaining 14 patients was attributed to the strict requirements regarding the dosage of steroid use and the necessity for positive LRT cultures as an entry criterion (Table 3).

### The proposed criterion for IPA in critically Ill COPD patients admitted to the ICU

Modified criteria for defining probable IPA based on the Bulpa criteria are proposed mainly based a revised interpretation of the requirements regarding microbiological findings (Table 4).

**Table 4:** Modified Bulpa criteria for probable IPA in critically Ill COPD patients admitted to an ICU

Criteria for diagnosing probable IPA in critically ill COPD patients admitted to ICU
1. Host factors ( the following at the same time)
i) Pulmonary function: A level of GOLD III or IV
ii) Steroid: treated with systemic steroid use, with no specific requirement regarding dose or course; or treated with an inhaled steroid for at least 3 months
2. Compatible Signs or Symptoms (one of the following)
i) Fever refractory to at least three days of appropriate antibiotic therapy ii) Recrudescent fever after a period of defervescence at least 48 h while still on antibiotics and without other apparent cause iii) Recent dyspnea or hemoptysis iv) Worsening respiratory insufficiency despite appropriate therapy and ventilator support
3. Radiological Findings (one of the following)
Abnormal imaging on CT or X-ray, within three month i) Nonspecific infiltrates and patchiness ii) Multiple nodules distributed along the airway ii)Well- shaped nodule(s), with or without halo sign iv)Wedge-shaped consolidation v) Mass consolidation vi) Air-crescent sign vii) Cavitation
4. Mycological Data (one of the following)
i) Positive culture and/or microscopy for *Aspergillus* from LRTs ii) Two consecutive positive serum/BALF GM tests^*,#^ iii) One positive BALF test and one positive serum GM test

^*,#^: A serum galactomannan test greater than 0.5 ng/ml and BALF galactomannan test greater than 0.8 ng/ml were defined as a positive result

Note: The diagnose of probable IPA could be made when a critically ill COPD patients in ICU with a pulmonary functional level of GOLD III or IV, a history of steroid use for at least 3 months and appropriate compatible signs or symptoms, having any major radiological sign of pneumonia and one of the microbiological findings.

*IPA* Invasive Pulmonary Aspergillus; *COPD* Chronic Obstructive Pulmonary Disease; *ICU* Intensive Care Unit; *GOLD* Global Initiative for Chronic Obstructive Lung Disease; *CT* Computed Tomography; *LRT* Lower Respiratory Tract; *BALF* Bronchoalveolar Lavage Fluid; *GM* Galactomannan

Probable IPA can be diagnosed when a critically ill COPD patient in the ICU exhibits a pulmonary functional level of GOLD III or IV, a history of steroid use of at least 3 months (irrespective of administration route and dose/ course) and appropriate compatible signs or symptoms, the presence of any major radiological sign of pneumonia and one of the mentioned microbiological findings.

## Discussion

To the best of our knowledge, the current study is the first to compare the validity of diagnosis between the existed three criteria commonly used for IPA. The major power of this study was that we evaluated the “diagnostic rate” of probable IPA in different populations, and validated the results based on a proven population.

The major finding of this study is that we verified that Bulpa criteria was the most suitable criteria for IPA diagnosis in critically ill COPD patients admitted to ICU compared with the other two. While the defects in microbiological findings still existed in Bulpa criteria, the modified Bulpa criteria for probable IPA were proposed and will need further verification by comparing against proven IPA cases in the future studies. Furthermore, We analyzed reasons for the highest “diagnostic rate” in Bulpa criteria.

We found that many non-classified patients by EORTC/ MSG and ICU criteria could be diagnosed as probable IPA by Bulpa criteria. Bulpa criteria made less strict requirements in dose/ course of steroid and typical CT manifestations, thus improving the“ diagnostic rate”. As reported, almost 30-70% of IPA patients in an ICU were not in a severely immunocompromised condition [16, 17]. Most COPD patients lack the host factors that are required by the EORTC/ MSG and ICU criteria with the exception of steroid usage. In addition, although precise doses and courses of steroid cannot be extrapolated from the literature for COPD patients, data support the fact that patients with underlying lung diseases are at risk for IPA at much lower doses and shorter courses [1, 4, 5, 18–21]. Recently, some reports have suggested that high doses of inhaled corticosteroids may also be a risk factor for IPA [22–24], and some COPD patients might develop IPA even without exposure to steroids [25]. Thus, in the clinical practice, there is no need for the excessively strict requirements regarding the dose and course of systemic steroid use, and the inhaled steroid should also be contained.

Nonspecific manifestations, such as patchiness or consolidations, are more frequently in COPD patients. The research of Meersseman W [26] and Vandewoude K [9] reported that the halo signs only appeared in 5-17% cases of COPD that are complicated with IPA. Our research [27] also demonstrated that patchiness (76.2%) were the most common CT sign among IPA in critically ill COPD patients admitted to ICU, while the angio-invasive patterns (including halo sign, wedge consolidation and air-crescent sign/ cavity) had a relatively low percent. Therefore, there is no need for the excessively strict requirements in typical CT manifestations as well in diagnostic criteria for IPA in critically ill COPD patient.

Defects in microbiological findings existed in Bulpa criteria. The lack of BALF GM tests in mycological data was the deficiency of the Bulpa criteria, which might underestimate some IPA patients. Our previous study [15] showed that positive BALF GM was observed earlier than that in LRT secretion culture (1 day versus 3.8 days), and an appropriate cut-off value enabled the discrimination of infection from colonization with *Aspergillus*. Therefore, the two consecutive positive BALF GM tests were found useful in diagnosing cases of probable IPA, and should be added into Bulpa criteria. Besides, positive serum antibody test for *A. fumigatus* (including precipitins) is included in the Bulpa criteria; however, it takes at least 3-6 months to elicit an antibody response [8, 28, 29], and the course of acute IPA is too rapid compared to antibody formation. Moreover, the antibody response is often weak in severe COPD patients on long-term steroid therapy [8]. Serum antibody testing for *A. fumigatus*, including that based on precipitins is an extremely useful technique in patients with chronic pulmonary aspergillosis (CPA) [8, 29, 30], and the use of this test in clinical practice is far from universal, so it should be removed from the Bulpa criteria.

We also noticed that the semi-quantitative *Aspergillus*-positive culture of BALF, without bacterial growth but with a positive cytological smear showing branching hyphae, is included in the ICU criteria as an alternative item for host factors; nevertheless, mixed infections with bacteria are common in critically ill COPD patients in the ICU; therefore, it was not appropriate to add it to Bulpa criteria as an alternative item for host factors.

There were several limitations in our study. The greatest one was the small number (only 5) of proven IPA cases in our study, for which histopathological specimens were available to verify the validity of the clinical assumption, thus we could only estimate the “diagnostic rate” of probable IPA cases in population one, two and three, and observed the consistency of the results with those for the proven IPA group, leading to weak persuasiveness. Moreover, it may also have a problem in overestimating the number of IPA based on Bulpa criteria; however, a delayed diagnosis was associated with a worse prognosis, we would rather accept a diagnose criteria with a lower missed diagnostic rate. Third, the sample size was relatively small because of the strict inclusion criteria, which included critically ill COPD patients in an ICU with a pulmonary functional level of GOLD III or IV, and the availability of CTs obtained within a short period. The above limitations require further investigations.

## Conclusion

Among the existing three criteria, the Bulpa criteria are the most suitable for diagnosing probable IPA in critically ill COPD patients admitted to ICU. The modified Bulpa criteria for probable IPA was proposed and needs further confirmation,

## Additional file

Additional file 1: “Three Key Factors” of the Included Patients. Note: The history of steroid use, abnormal radiology and mycological findings of the included 59 COPD patients were listed in Additional file 1, and patients were diagnosed as probable IPA using three criteria respectively. “Y” means that the case was could be diagnosed as probable/ putative IPA according to the criteria; “N” means that the case was could not be diagnosed as probable/ putative IPA according to the criteria. (DOCX 128 kb)

## Abbreviations

(R)ICU: (Respiratory) Intensive care unit
BALF: Bronchoalveolar lavage fluid
COPD: Chronic obstructive pulmonary disease
CPA: Chronic pulmonary aspergillosis
CT: Computed tomography
CTD: Connective tissue disease
CXR: Chest X ray
EORTC/ MSG: European Organization for the Research and Treatment of Cancer/Invasive Fungal Infections Cooperative Group
ETA: Endotracheal aspiration
GM: Galactomannan
GOLD: Global initiative for chronic obstructive lung disease
HIV: Human immunodeficiency virus
HSCT: Haematopoietic stem cell transplantation
IPA: Invasive pulmonary aspergillosis
LRT: Lower respiratory tract
SOT: Solid organ transplantation
