# Supplementary material for: Is Bulpa criteria suitable for the diagnosis of probable invasive pulmonary Aspergillosis in critically ill patients with chronic obstructive pulmonary disease? A comparative study with EORTC/ MSG and ICU criteria
Source: BMC Infect Dis. 2017 Mar 14;17:209. doi: 10.1186/s12879-017-2307-y (PMC5351185; doi:10.1186/s12879-017-2307-y)
Supplement: Additional file 1: — “Three Key Factors” of the Included Patients. Note: The history of steroid use, abnormal radiology and mycological findings of the included 59 COPD patients were listed in Additional file 1, and patients were diagnosed as probable IPA using three criteria respectively. “Y” means that the case was could be diagnosed as probable/ putative IPA according to the criteria; “N” means that the case was could not be diagnosed as probable/ putative IPA according to the criteria. (DOCX 128 kb) [file 12879_2017_2307_MOESM1_ESM.docx]

**“Three Key Factors” of the Included Patients**

|  | **Host Factors** | **Typical Manifestations on CTs** | **Mycological criteria** | **EORTC** | **Bulpa** | **ICU** |
| --- | --- | --- | --- | --- | --- | --- |
| 1 | Intravenous and oral, 16.7*12 | Multiple nodules, wedge-shaped consolidation | Serum GM: 0.503,1.321 | N | Y | N |
| 2 | Intravenous, oral, inhaled, 18*27 | Multiple nodules, halo sign | A. fumigatus (ETA*3,Spu*6) | Y | Y | N |
| 3 | Intravenous and inhaled, 21.5*23 | Air crescent sign, halo sign | A. fumigatus (Spu*3) | Y | Y | Y |
| 4 | Intravenous and orals, 38.3*10 | Abnormal, none of the typical signs | A. fumigatus (Spu*1), serum GM: 0.853, 1.153 | N | Y | Y |
| 5 | Intravenous and orals, 27.4*17 | Cavity | A. fumigatus (Spu*5), BALF GM: 7.128 | N | Y | Y |
| 6 | Intravenous, 15*3 | Abnormal, none of the typical signs | BALF GM: 0.932, 1.872 | N | N | N |
| 7 | Intravenous, 40*3 | Abnormal, none of the typical signs | A. fumigatus (Spu*2), serum GM: 0.726 | N | Y | Y |
| 8 | Intravenous and oral, 33.3*6 | Cavity, halo sign, multiple nodules | A. fumigatus (ETA*1), serum GM: 1.081, 2.032 | N | Y | Y |
| 9 | Inhaled steroids | Abnormal, none of the typical signs | A. fumigatus (Spu*7), BALF GM: 1.305 | N | Y | N |
| 10 | Intravenous, oral, steroids, 22.1*23 | Abnormal, none of the typical signs | 1. fumigatus (ETA*8), A. flavus (ETA*1,BALF*1) | N | Y | Y |
| 11 | Inhaled | Abnormal, none of the typical signs | A. niger (spu*3) | N | Y | N |
| 12 | Inhaled and intravenous, 40*1 | Abnormal, none of the typical signs | none | N | N | N |
| 13 | Inhaled | Abnormal, none of the typical signs | A. fumigatus (Spu*5), serum GM: 1.006 | N | Y | N |
| 14 | Intravenous and oral, 16.5*22 | Multiple nodules, wedge-shaped consolidation | A. niger (Spu*5), serum GM: 0.656 | Y | Y | N |
| 15 | Inhaled | Abnormal, none of the typical signs | none | N | N | N |
| 16 | Inhaled | Abnormal, none of the typical signs | none | N | N | N |
| 17 | Inhaled | Abnormal, none of the typical signs | none | N | N | N |
| 18 | No history of steroid use | Abnormal, none of the typical signs | none | N | N | N |
| 19 | Inhaled | Multiple nodules | BALF GM: 0.942, 1.115 | N | N | N |
| 20 | Inhaled | Abnormal, none of the typical signs | none | N | N | N |
| 21 | No history of steroid use | Multiple nodules, cavity | none | N | N | N |
| 22 | No history of steroid use | Wedge-shaped consolidation | none | N | N | N |
| 23 | No history of steroid use | Abnormal, none of the typical signs | Serum GM: 0.621, 0. 399 | N | N | N |
| 24 | No history of steroid use | Abnormal, none of the typical signs | none | N | N | N |
| 25 | No history of steroid use | Abnormal, none of the typical signs | BALF GM: 0.812, 0.689 | N | N | N |
| 26 | No history of steroid use | Abnormal, none of the typical signs | none | N | N | N |
| 27 | No history of steroid use | Multiple nodules | none | N | N | N |
| 28 | Inhaled | Abnormal, none of the typical signs | none | N | N | N |
| 29 | Inhaled and oral, 5*3 | Cavities, wedge-shaped consolidation | BALF GM: 1.193, 1.224 | N | N | N |
| 30 | Intravenous, 40*5 | Abnormal, none of the typical signs | Serum GM: 0.520, 0.664 | N | Y | N |
| 31 | Inhaled | Cavities | none | N | N | N |
| 32 | No history of steroid use | Abnormal, none of the typical signs | none | N | N | N |
| 33 | No history of steroid use | Abnormal, none of the typical signs | none | N | N | N |
| 34 | No history of steroid use | Multiple nodules, cavities | none | N | N | N |
| 35 | No history of steroid use | Abnormal, none of the typical signs | none | N | N | N |
| 36 | Inhaled | Abnormal, none of the typical signs | none | N | N | N |
| 37 | Inhaled | Abnormal, none of the typical signs | none | N | N | N |
| 38 | Intravenous, oral 21.7*14 | Multiple nodules | BALF GM 3.17, 3.04 | N | N | Y |
| 39 | Inhaled | Abnormal, none of the typical signs | none | N | N | N |
| 40 | No history of steroid use | Abnormal, none of the typical signs | none | N | N | N |
| 41 | No history of steroid use | Abnormal, none of the typical signs | none | N | N | N |
| 42 | No history of steroid use | Abnormal, none of the typical signs | none | N | N | N |
| 43 | No history of steroid use | Abnormal, none of the typical signs | none | N | N | N |
| 44 | No history of steroid use | Single nodule | none | N | N | N |
| 45 | No history of steroid use | Multiple nodules with halo signs | none | N | N | N |
| 46 | No history of steroid use | Abnormal, none of the typical signs | none | N | N | N |
| 47 | Inhaled | Abnormal, none of the typical signs | none | N | N | N |
| 48 | Inhaled | Abnormal, none of the typical signs | none | N | N | N |
| 49 | No history of steroid use | Abnormal, none of the typical signs | none | N | N | N |
| 50 | Inhaled | Abnormal, none of the typical signs | none | N | N | N |
| 51 | Inhaled | Multiple nodules | none | N | N | N |
| 52 | Intravenous, oral, inhaled, 31*10 | Mass consolidation, halo sign | A. fumigatus (ETA*4,BALF*2),  A. flavus (ETA*7, BALF*2), positive biopsy | N | Y | Y |
| 53 | Oral, inhaled, 20*2 | Abnormal, none of the typical signs | A. fumigatus (Spu*2) | N | Y | Y |
| 54 | Inhaled | Abnormal, none of the typical signs | none | N | N | N |
| 55 | Intravenous, oral, 35.6*9 | Wedge-shaped consolidation, multiple nodules | A. fumigatus (Spu*3, BALF*1), positive biopsy | N | Y | Y |
| 56 | Inhaled | Abnormal, none of the typical signs | A. fumigatus (ETA*3,BALF*1), positive biopsy | N | Y | N |
| 57 | Intravenous, oral, inhaled, 18*30 | Multiple nodules | A. fumigatus (Spu*3), serum GM: 2.343, 2.242  Positive biopsy | Y | Y | N |
| 58 | Inhaled | Abnormal, none of the typical signs | A.fumigatus (ETA*6, BALF*2), serum GM: 0.817  Positive biopsy | N | Y | N |
| 59 | Inhaled | Abnormal, none of the typical signs | A. fumigatus (Spu*2, BALF*1) | N | Y | N |

Note: “Three key factors (the history of steroid use, abnormal radiology and mycological findings)” of the included 59 COPD patients were listed above, and patients were diagnosed as probable IPA using three criteria respectively.

“Y” means that the case was could be diagnosed as probable/ putative IPA according to the criteria; “N” means that the case was could not be diagnosed as probable/ putative IPA according to the criteria.

The steroid doses were converted to prednisone dose, and presented as mg*day.

BALF: Bronchoalveolar Lavage Fluid; ETA: endotracheal aspiration; GM: Galactomannan; Spu: Sputum.
